# High-Density Lipoproteins and Cardiovascular Disease: The Good, the Bad, and the Future

**DOI:** 10.3390/ijms22147488

**Published:** 2021-07-13

**Authors:** Josep Julve, Joan Carles Escolà-Gil

**Affiliations:** 1 Institut d’Investigacions Biomèdiques IIB Sant Pau, 08041 Barcelona, Spain; 2 CIBER de Diabetes y Enfermedades Metabólicas Asociadas (CIBERDEM), 28029 Madrid, Spain; 3 Departament de Bioquímica i Biologia Molecular, Universitat Autònoma de Barcelona, 08041 Barcelona, Spain

Epidemiological, clinical, and experimental studies have shown that low levels of plasma high-density lipoprotein cholesterol (HDL-C) are associated with increased atherosclerotic cardiovascular disease (CVD). Nevertheless, HDL-targeted drugs, such as cholesteryl ester transfer protein inhibitors, fibrates, and niacin, have failed to reduce cardiovascular events in large-scale randomized controlled trials. Furthermore, no plausible causal link between HDL-C and CVD risk was found in Mendelian randomization studies. These data strongly indicate that increased HDL-C levels do not always correlate with enhanced beneficial HDL properties, thus questioning the potential of HDL-C as a biomarker of HDL function. We are pleased to introduce this special issue, “High-Density Lipoproteins and Cardiovascular Disease: The Good, the Bad, and the Future”, which aims to present timely and informative findings on the role of HDL functions, their influence on CVD and other non-cardiovascular diseases, and on the development of new HDL-based therapeutic strategies that optimize HDL functions.

HDL is a highly heterogeneous particle and carries a large variety of lipids, proteins, hormones, vitamins, and miRNAs which confer HDL particles with multiple cardioprotective functions. These include the enhancement of macrophage reverse cholesterol transport and endothelial functions, antioxidant, anti-inflammatory, anti-thrombotic and anti-apoptotic properties, and also antidiabetic and immunomodulatory properties (reviewed in [1,2]). The different compositions of HDL subpopulations are related to their functions, but the assignment of specific molecules to HDL functions remains largely poorly understood. Sphingosine 1-phosphate (S1P) is a bioactive molecule, mainly bound to HDL, that is thought to be beneficial in the occurrence and development of myocardial ischemia (reviewed in [3]). Furthermore, S1P metabolism appears to be unbalanced in cardiometabolic diseases such as obesity and diabetes mellitus [3]. S1P-enrichment of HDL might constitute a novel strategy in the treatment of cardiometabolic complications.

HDL metabolism is also relevant to non-cardiovascular diseases. Obesity significantly alters HDL metabolism, resulting in altered HDL subclass distribution, composition, and function through multiple mechanisms (reviewed in [4]). These findings motivate further research on the potential effects of anti-obesity treatments on HDL functions and their physiopathological consequences. Recent findings also revealed a significant impact of HDL on pulmonary artery vasoreactivity and an improved prognosis in patients with pulmonary arterial hypertension (reviewed in [5]). The mechanism by which HDL exerts its protective effect in pulmonary circulation remains largely unknown. Recent evidence indicates a role of HDL in Alzheimer disease. Apolipoprotein (apo) E is mainly bound to HDL in the cerebrospinal fluid and current evidence highlights the importance of apoE isoforms in modulating the pathogenesis of Alzheimer’s disease (reviewed in [6]), thereby suggesting that novel apoE-based strategies could prevent or ameliorate Alzheimer’s disease. Furthermore, the association between low HDL cholesterol and CVD can be further confounded by chronic kidney disease (CKD). HDL properties are impaired in this disease, resulting in increased CVD risk (reviewed in [7]). 

Beyond these review articles, several original articles of this special issue moved towards the identification of specific HDL molecules that can alter HDL functions in pre-clinical and translational studies and their therapeutic utility. Therefore, apoB-depleted plasma was effective in suppressing Aβ accumulation in bioengineered vessels, preventing Aβ fibrillization, and suppressing TNFα-induced vascular inflammation [8]. Furthermore, isolated HDL further suppressed Aβ-induced vascular inflammation, improved Aβ vascular clearance and induced endothelial NO production [8] which is relevant to Alzheimer’s disease progression. We also found that overexpression of human apoA-I in severe obese leptin receptor-deficient mice enhanced the main HDL anti-atherogenic properties while exacerbating weight gain and fatty liver disease [9]. These adverse metabolic side effects might raise concerns regarding the use of apoA-I-based therapies in obese humans. Another report demonstrated significant risk associations for elevated urinary albumin excretion and for multiple HDL-associated parameters, as well as significant interactions of elevated urinary albumin excretion with apoA-I/HDL particles and with concentration of medium size HDL particles [10], thereby indicating that HDL particles share pathogenic pathways with elevated urinary albumin excretion in CVD risk. In this context, HDL remodeling was also found to be highly affected in CKD patients in which plasma levels of nascent preβ1-HDL were significantly elevated in non-dialyzed patients with advanced stages of CKD [11]. Another report demonstrated that reconstituted HDL with apoA-I Milano, an apoA-I mutant resulting from an arginine 173 to cysteine mutation with antiatherogenic properties, reversed pathological remodeling and cardiac dysfunction and normalized wet lung weight in a mouse model of diabetic cardiomyopathy induced by a high-sugar/high-fat diet [12]. In contrast, the lipoprotein lipase inhibitor nordihydroguaiaretic acid increased small HDL particles and worsened obesity and metabolic complications in leptin receptor-deficient mice fed a Western-type diet [13]. In this context, patients with carotid plaques showed higher triglyceride-containing HDL, which was associated with metabolic and arteriosclerotic vascular alterations [14], thereby indicating that HDL-triglycerides should be considered a biomarker of metabolic and cardiovascular risk.

An interesting mechanistic work revealed that cholesterol acceptors from macrophage lipids during the cholesterol efflux process, particularly apoA-I, were positive regulators of various pro-atherogenic lipid species, such oxysterols, sphingomyelins and ceramides [15]. This latter work also indicates that apoA-I function is not only limited to the efflux of cholesterol and phospholipids and it could be a major regulator of the foam cell lipidome, playing a critical role in reducing lipid species involved in atherogenesis. Other findings of this special issue demonstrated that bisphenol A promoted atherosclerosis in low-density lipoprotein (LDL) receptor-deficient mice, at least in part, by activating NF-κB, which downregulates apoA-I gene expression and leads to lower HDL levels [16]. In contrast, pretreatment of apoE-deficient mice with HDL decreased serum amyloid A (SAA) pro-inflammatory activity, inhibited SAA-mediated enhancement of aortic atherosclerosis and renal function, and prevented changes to the glomerular Bowman’s space [17].

The importance of the HDL lipidome was recently revealed in a report that demonstrated that patients with type 2 diabetes mellitus and normal serum lipid profiles, even at diagnosis, showed significant alterations in lipid HDL composition which were qualitatively similar to those found in normolipidemic patients with established coronary heart disease [18]. This study also reinforces the concept that nuclear magnetic resonance-based lipidomics allow the gain of pathophysiological knowledge, evaluation and/or discovery of novel disease biomarkers. The need for further research in this field was also showed in a recent work in which endothelial lipase (EL) overexpression in mice significantly decreased serum HDL-C levels but unexpectedly increased the content of its main antioxidant enzyme, paraoxonase 1 (PON1), and its activity [19]. However, EL serum levels were not significantly correlated with HDL levels in humans, whereas HDL PON1 content was positively associated with EL serum levels [19]. Furthermore, EL-induced alterations of the HDL lipidome were not related to HDL PON1 content. This point deserves further investigation.

Finally, we hope that these articles contributed by experts of this field will provide valuable resources to researchers working on HDL functional alterations and the development of HDL-based therapies to treat diseases related to HDL.
